# Effect of L‐Citrulline Intake on Blood Pressure in Cold Environments: A Systematic Review and Meta‐Analysis of Randomized Controlled Trials

**DOI:** 10.1002/fsn3.71603

**Published:** 2026-03-06

**Authors:** Ping Luo, Jingfeng Chen, Kang Liu, Jia Zhang

**Affiliations:** ^1^ School of Physical Education Wuhan Sports University Wuhan China; ^2^ Department of Health and Physical Education The Education University of Hong Kong Hong Kong China; ^3^ School of Physical Education and Health Nanning Normal University Nanning China; ^4^ School of Physical Education Chongqing University Chongqing China

**Keywords:** blood pressure, cold environment, diastolic blood pressure, L‐citrulline, systolic blood pressure

## Abstract

The primary objective of this systematic review and meta‐analysis is to investigate whether L‐citrulline supplementation can counteract the adverse effects of cold environments on individual blood pressure (BP), providing scientific evidence for the clinical development and application of L‐citrulline as a cardiovascular protective nutritional supplement. A comprehensive search was conducted across four electronic databases: PubMed, Cochrane Library, Embase, and Web of Science. The search period was limited from database inception to May 28, 2025. The Cochrane Risk of Bias tool and JADAD scoring scale were used to assess risk of bias and literature quality of the included randomized controlled trials (RCTs). Statistical analysis of BP data was performed using RevMan 5.4.1 software, employing both random‐effects and fixed‐effects models for data analysis, and forest plots were generated. The overall intervention effect was evaluated using the weighted mean difference (WMD) and its 95% confidence interval (CI). A total of 6 RCTs investigating the effects of L‐citrulline intake on BP in cold environments were included, involving 162 participants (intervention group: 87; control group: 75). Results indicate that L‐citrulline intake significantly reduced cold‐induced SBP (−9.28 mmHg [95% CI: −10.66 to −7.90], *p* < 0.001) and DBP (−5.33 mmHg [95% CI: −9.38 to −1.27], *p* = 0.01). Subgroup analysis revealed significant reductions in brachial SBP (−8.74 mmHg [95% CI: −10.61 to −6.88], *p* < 0.001), aortic SBP (−9.93 mmHg [95% CI: −11.98 to −7.88], *p* < 0.001), and aortic DBP (−5.60 mmHg [95% CI: −9.56 to −1.64], *p* < 0.001). However, brachial DBP reduction did not reach statistical significance but showed a trend toward decrease (−5.10 mmHg [95% CI: −11.71 to 1.52], *p* = 0.13). Meta‐analysis results indicate that L‐citrulline supplementation can significantly improve cold exposure‐induced BP elevation, providing scientific evidence for the clinical development and application of cardiovascular protective nutritional supplements.

## Graphical abstract

This study investigated the effect of L‐citrulline supplementation on BP under cold exposure and found that it significantly reduced both SBP and DBP, thereby alleviating cold‐induced hypertension, thus providing a scientific basis for cardiovascular protection. Future studies should further investigate its optimal dosage.

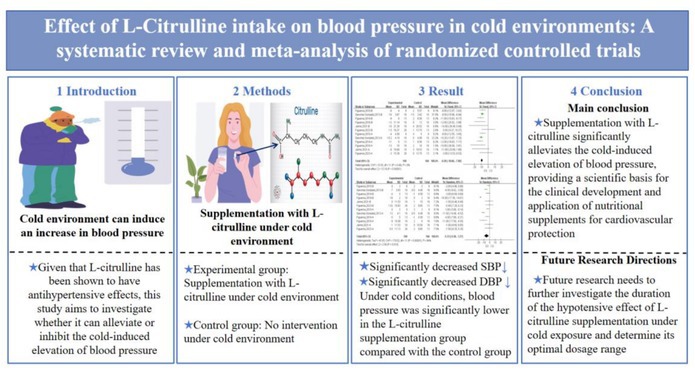

**Abbreviations**
BP: blood pressure
CI: confidence interval
CPT: cold pressor test
CVD: cardiovascular disease
DBP: diastolic blood pressure
eNOS: endothelial nitric oxide synthase
mmHg: millimeters of mercury
NO: nitric oxide
PICOS: population, intervention, comparison, outcomes, study design
PRISMA: preferred reporting items for systematic reviews and meta‐analyses
PROSPERO: international prospective register of systematic reviews
RCT: randomized controlled trial
RoB 2: Cochrane risk of bias tool (version 2)
SBP: systolic blood pressure
WMD: weighted mean difference

## 1 Introduction

Cardiovascular disease (CVD) represents the leading cause of the global disease burden (Chong et al. 2024; GBD 2021 ASEAN Cardiovascular Diseases Collaborators 2025; Roth et al. 2020). Research indicates that in 2019, approximately 18.6 million lives were lost worldwide due to CVD (Roth et al. 2020). The development of CVD is influenced by multiple factors, including genetic predispositions (Larsson et al. 2023), unhealthy lifestyles (Lv et al. 2017), and environmental factors (Khraishah et al. 2022). Numerous studies have confirmed that ambient temperature significantly impacts cardiovascular health, with both high and low temperatures potentially increasing CVD risks (Achebak et al. 2024; Chen et al. 2022; Stewart et al. 2017; Yang et al. 2015). In low‐temperature environments, the human body undergoes peripheral vasoconstriction, most prominently manifested as elevated blood pressure (BP) (Sun et al. 2023; Xu et al. 2019, 2024). A meta‐analysis including 14 studies demonstrated that a 1°C decrease in outdoor average temperature correlates with a 0.26 mmHg increase in systolic blood pressure (SBP) and a 0.13 mmHg increase in diastolic blood pressure (DBP) (Wang et al. 2017). This temperature‐related BP variation is particularly pronounced during seasonal transitions, especially in regions with extreme seasonal temperature differences. A large cohort study involving 23,000 CVD patients demonstrated that the mean winter SBP (145 mmHg) was significantly higher than that in summer (136 mmHg), with SBP increasing by 6.2 mmHg for every 10°C decrease in temperature. More importantly, this BP elevation was closely associated with adverse outcomes, with cardiovascular mortality risk increasing by 21% for every 10 mmHg increase in SBP, and winter cardiovascular mortality rates being 41% higher than those in summer (Yang et al. 2015). Given that cold environment‐induced BP elevation is one of the important modifiable risk factors for CVD (Gu et al. 2018; Yokoyama et al. 2014), targeted interventions to control elevated BP during cold weather are particularly crucial (Xu et al. 2019, 2024). This approach not only enhances individual health outcomes but also significantly reduces the burden on public health systems (Bruno and Taddei 2015; Yu et al. 2020).

Multiple studies have confirmed that cold environments can lead to increased BP in humans (Duranton et al. 2018; Lanzinger, Hampel, et al. 2014; Modesti 2013), but the underlying temperature‐BP association mechanism has not been fully elucidated (Osborn and Dailey‐Krempel 2025; Xu et al. 2019). Research indicates that cold environments elevate BP through multiple mechanisms. Cold exposure can activate the sympathetic nervous system (Jdidi et al. 2024; Leppäluoto et al. 2001; Yoshimoto et al. 2025) and the renin‐angiotensin‐aldosterone system (Sun et al. 2003; Yoshimoto et al. 2025), leading to peripheral vasoconstriction and increased heart rate, which subsequently raises BP (Jdidi et al. 2024; Leppäluoto et al. 2001; Sun et al. 2003; Yoshimoto et al. 2025). On the other hand, cold can upregulate L‐type calcium channels (Saad et al. 2008), induce oxidative stress (Briones and Touyz 2010; Martarelli et al. 2011), and impair endothelial NO bioavailability (Ding et al. 2020; Lanzinger, Breitner, et al. 2014), resulting in impaired vasodilatory function and increased BP. Additionally, low temperatures reduce sodium excretion, leading to sodium retention and increased blood volume, which raises BP (Brook et al. 2011). L‐citrulline, as a non‐essential amino acid, has been demonstrated by multiple studies to promote the synthesis of nitric oxide (NO) (Holguin et al. 2019; Wileman et al. 2003), which plays a vital role in vasodilation (Leo et al. 2021; Vallance and Chan 2001). Existing meta‐analyses also indicate that L‐citrulline supplementation helps improve BP in adults (Barkhidarian et al. 2019; Luo, Chen, et al. 2025; Luo, Ziyi, et al. 2025; Mahboobi et al. 2021; Yang et al. 2019). Research has found that oral L‐arginine supplementation can attenuate the reactivity of brachial artery BP to cold stress in men with hypercholesterolemia (West et al. 2005). However, L‐citrulline, as a precursor to L‐arginine, possesses unique metabolic advantages in the NO synthesis pathway, with superior bioavailability compared to direct L‐arginine supplementation (El‐Bassossy et al. 2012; Schwedhelm et al. 2008). Based on this evidence, L‐citrulline may have the potential to counteract cold‐induced BP elevation, thereby enhancing vascular function, reducing BP, and alleviating the cardiovascular burden of cold exposure. However, there is currently a lack of systematic reviews and meta‐analyses specifically examining the effects of L‐citrulline supplementation on BP in cold environments, and relevant research remains limited. Thus, further studies are needed to validate L‐citrulline's BP‐regulating efficacy in cold conditions to confirm its effectiveness in BP modulation.

Therefore, the primary objective of this systematic review and meta‐analysis is to investigate whether L‐citrulline supplementation can counteract the adverse effects of cold environments on individual BP, providing scientific evidence for the clinical development and application of L‐citrulline as a cardiovascular protective nutritional supplement.

## 2 Materials and Methods

### 2.1 Protocol Registration

This systematic review and meta‐analysis rigorously adheres to the PRISMA (Preferred Reporting Items for Systematic Reviews and Meta‐Analyses) reporting guidelines (Page et al. 2021). Prior to study commencement, the protocol was registered on the PROSPERO online platform under the following registration number: CRD420251061935.

### 2.2 Literature Search Strategy

A comprehensive search was conducted across four electronic databases: PubMed, Cochrane Library, Embase, and Web of Science. The search period was limited from database inception to May 28, 2025. In PubMed and Cochrane Library, MeSH term searches were performed for “citrulline” “cold temperature” and “blood pressure” to identify relevant search terms. Simultaneously, in the Embase database, Emtree term searches were conducted for “citrulline,” “cold,” and “blood pressure.” The relevant search terms are as follows: (“citrulline” OR “citrulline malate” OR “l‐citrulline”) AND (“cold temperature” OR “chill temperature” OR “low temperature” OR “cold” OR “chilly” OR “frigid” OR “cold conditions” OR “frigid conditions” OR “icy conditions” OR “cold pressor test” OR “CPT”) AND (“blood pressure” OR “BP” OR “diastolic blood pressure” OR “DBP” OR “systolic blood pressure” OR “SBP” OR “high blood pressure” OR “HBP” OR “elevated blood pressure” OR “hypertension” OR “hypertensive” OR “blood pressure reduction” OR “rest blood pressure” OR “mean blood pressure” OR “blood pressure monitoring” OR “BPM” OR “ambulatory blood pressure” OR “ABP” OR “ambulatory blood pressure monitoring” OR “ABPM” OR “automated office blood pressure” OR “AOBP” OR “home blood pressure monitoring” OR “HBPM” OR “blood pressure management” OR “resting blood pressure” OR “daytime blood pressure” OR “nighttime blood pressure” OR “24 hours blood pressure” OR “24‐h blood pressure”) AND (“random” OR “randomized” OR “randomly” OR “randomized” OR “randomized controlled trial” OR “RCT”). The literature screening process was conducted independently by two researchers (PL and JFC). During the literature screening process, published relevant reviews were examined to ensure the comprehensiveness of the search. The detailed documentation of the literature search process is provided in Appendix S1.

### 2.3 Inclusion Criteria

According to the PICOS principle, retrieved literature will be included if it meets the following criteria: (1) Population: adults aged 18 years and above; (2) Intervention: the experimental group receives L‐citrulline supplementation alone or L‐citrulline combined with other nutritional supplements under cold stress or low‐temperature environments; (3) Comparison: the control group receives a placebo under the same cold stress or low‐temperature environments; (4) Outcomes: measurement data for brachial artery blood pressure and/or aortic blood pressure before and after intervention; (5) Study design: only RCTs are included.

### 2.4 Exclusion Criteria

Studies will be excluded if they meet any of the following criteria:(1)Non‐RCT interventions; (2) Non‐English publications; (3) Duplicate publications; (4) Intervention duration less than 1 week.

### 2.5 Literature Risk of Bias and Quality Assessment

The risk of bias for included RCTs was assessed using the Cochrane Risk of Bias tool (RoB 2) (Sterne et al. 2019). The quality of the literature was evaluated using the JADAD scoring scale (Jadad et al. 1996). The JADAD scoring method evaluates studies based on random sequence generation, allocation concealment, blinding, and description of participant withdrawals or dropouts. Studies scoring 4–7 points were classified as high quality, while those scoring 1–3 points were deemed low quality.

To ensure the reliability of risk of bias and quality assessment, the evaluation process was independently conducted by two experienced researchers (PL and JFC). If disagreements arose and could not be resolved through discussion between the two, they were resolved through consultation with a third researcher (JZ).

### 2.6 Data Extraction

The entire data extraction process was performed independently by two researchers. Primary extractions included the mean values and standard deviations of key outcome measures (brachial BP and aortic BP) before and after intervention across all groups. When included studies reported data as standard errors or confidence intervals, data were converted to estimated standard deviations using Cochrane‐recommended methods. The formula for mean difference is:

$${\mathrm{MD}}_{\text{diff}}=M_{\text{post}}-M_{\mathrm{pre}}$$

where *M*_post_ and *M*_pre_ represent the post‐intervention and baseline outcome means, respectively (Cumpston et al. 2019; Higgins et al. 2022). The standard deviation conversion formula follows (Higgins et al. 2022):

$$\sqrt{\left(SD_{\mathrm{pre}}^{2}+SD_{\text{post}}^{2}\right)-\left(2\times \text{Corr}\times SD_{\mathrm{pre}}\times SD_{\text{post}}\right)}$$

with the correlation coefficient (Corr) set at 0.5 according to the Cochrane Handbook guidelines. To address the presence of multiple intervention groups within a single study, we combined similar intervention groups to avoid reusing shared control groups and conducted separate independent analyses for DBP and SBP, thereby ensuring the statistical independence of all included data. The pooling formulas are as follows: assuming subgroup A has a sample size of *N*_1_, a mean of *M*_1_, and a standard deviation of SD_1_; and subgroup B has a sample size of *N*_2_, a mean of *M*_2_, and a standard deviation of SD_2_. *N* is the pooled sample size, *N* = *N*_1_ + *N*_2_; *M* is the pooled mean, *M* = (*N*_1_·*M*_1_ + *N*_2_·*M*_2_)/(*N*_1_ + *N*_2_); and SD is the pooled standard deviation, calculated using the formula:

$$\mathrm{SD}=\sqrt{\frac{\left(N_{1}-1\right)SD_{1}^{2}+\left(N_{2}-1\right)SD_{2}^{2}+\frac{N_{1}N_{2}}{N_{1}+N_{2}}\left(M_{1}^{2}+M_{2}^{2}-2M_{1}M_{2}\right)}{N_{1}+N_{2}-1}}$$

Relevant primary information extracted included: (1) Basic literature details (first author, publication year, country, etc.); (2) Participant information (population characteristics, age, body mass index, etc.); (3) Intervention specifics (supplements administered, dosage, daily frequency, intervention duration, etc.). During extraction, any missing critical information or data were requested from the original authors via email.

### 2.7 Statistical Analysis

This study used RevMan 5.4.1 for statistical analysis of BP data and to generate forest plots. The overall intervention effect was evaluated using the weighted mean difference (WMD) and its 95% confidence interval (CI). Heterogeneity between studies was assessed via the *I* ^*2*^ statistic (Higgins et al. 2003): *I* ^2^ < 25% indicated low heterogeneity risk, 25%–75% moderate risk, and > 75% high heterogeneity risk. For *I* ^2^ ≥ 25%, a random‐effects model was used for data pooling; otherwise, a fixed‐effects model was applied to enhance result accuracy. Sensitivity analysis was performed by sequentially excluding individual studies to test the robustness of overall results. Finally, publication bias was visualized through funnel plots and quantified via Egger's regression test. Funnel plots generated with Stata 17.0 provided visual assessment (Peters et al. 2008), while Egger's test used *p*‐values to quantify bias (*p* < 0.05 indicated significant publication bias; Egger et al. 1997; Peters et al. 2008).

## 3 Results

### 3.1 Literature Screening

**FIGURE 1:**
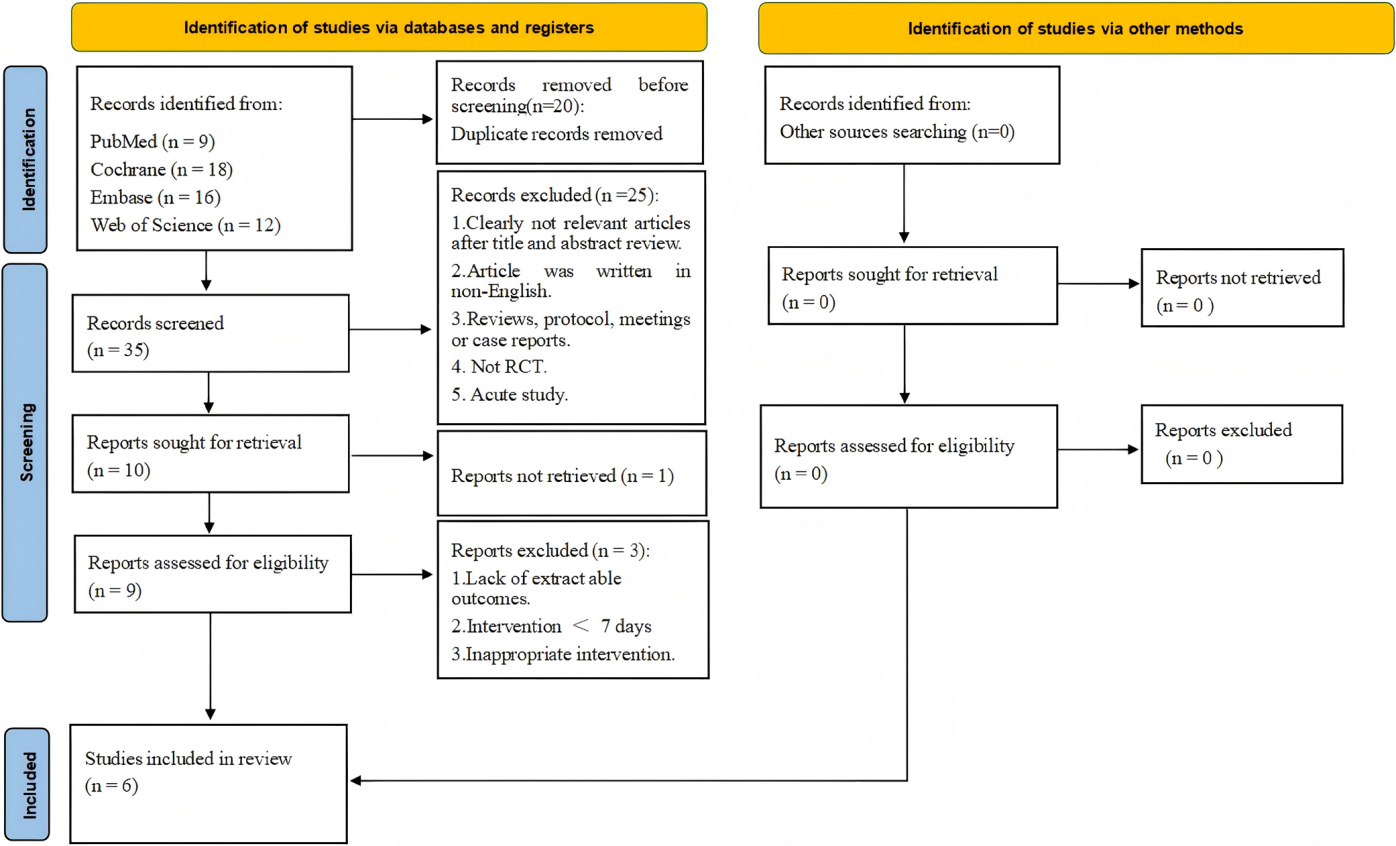
Flow diagram of systematic literature article search.

Initial searches across four major databases yielded 55 relevant articles. After screening, 6 RCTs investigating L‐citrulline supplementation's effects on BP in cold environments were included (Figueroa et al. 2016, 2023, 2010, 2014; Jaime et al. 2022; Sanchez‐Gonzalez et al. 2013). The search and screening process is summarized in Figure 1, with full documentation provided in Appendix S1.

### 3.2 Literature Characteristics

This systematic review incorporated 6 RCTs examining L‐citrulline supplementation effects on BP during cold exposure. All studies originated from United States institutions, collectively enrolling 162 participants (intervention group: *n* = 87; control group: *n* = 75). The characteristics of the literature are summarized in Table 1.

**TABLE 1:** Characteristics of the Literature.

First author, year	Country	Subject characteristics	Age (years)	Sample size (EG‐CG)	Body mass index (kg/m^2^)	Supplementary substance	Intervention plan (week/dose/Supplementary frequency/Testing process)	Outcome measure
Figueroa et al. (2010)	America	Young men	21.6 ± 0.9	8–9	25.7 ± 0.5	L‐Citrulline	4 weeks/3 g/2times/Immerse your right foot in cold water at 4°C for 2 min	①②
Sanchez‐Gonzalez et al. (2013)	America	Young men	23 ± 3	8–8	27.1 ± 1	L‐Citrulline	2 weeks/7–11 g/3times/Cold exposure of the environmental chamber to 4°C for 41 min. Cardiovascular measurements were taken during the subjects' resting state, 3‐min equal grip strength with 30% maximum voluntary contraction, and 3‐min post exercise period	①②
Figueroa et al. (2014)	America	Obese Hypertensive adults	57.4 ± 1.4	13–13	36.8 ± 2.1	L‐Citrulline + L‐Arginine	6 weeks/4 g of L‐Citrulline and 2 g of L‐Arginine/3times/Immerse the participant's right hand in water at 4°C for 2 min	①②
Figueroa et al. (2016)	America	Overweight men	24 ± 2	16–16	29.3 ± 1.1	L‐Citrulline	2 weeks/6 g/2times/The participant's left foot was passively immersed in 4°C ice‐cold water for 3 min	①②
Jaime et al. (2022)	America	Older adults	72.5 ± 7.3	16–16	26.7 ± 1.3	L‐Citrulline	2 weeks/6 g/2 times/The participant's left hand was passively immersed in 4°C water for 2 min while maintaining a prone position	①②
Figueroa et al. (2023)	America	Postmenopausal women	58 ± 4	26–13	29.0 ± 4.8	L‐Citrulline/L‐Citrulline + Glutathione	4 weeks/EG‐1 supplemented with 6 g L‐Citrulline, and EG‐2 supplemented with 2 g L‐Citrulline + 200 mg Aglutathione/2times/Participants immersed their right hand in 1°C–4°C cold water for 2 min	①②

*Note:* ①, brachial blood pressure; ②, aortic blood pressure.

Abbreviations: CG, control group; EG, experimental group; G, grams; MG, milligrams.

Participant characteristics revealed an age range of 21–73 years. Two trials involved healthy cohorts (Figueroa et al. 2010; Sanchez‐Gonzalez et al. 2013), while four focused on populations with established CVD or elevated CVD risk factors—specifically hypertensive (Figueroa et al. 2014), overweight (Figueroa et al. 2016), elderly (Jaime et al. 2022), and postmenopausal individuals (Figueroa et al. 2023). Notably, five studies included overweight participants (Figueroa et al. 2016, 2023, 2010; Jaime et al. 2022; Sanchez‐Gonzalez et al. 2013), and one enrolled obese subjects (Figueroa et al. 2014).

Regarding interventions, all trials measured pre‐ and post‐intervention brachial and aortic BP. L‐citrulline monotherapy was administered in four trials (Figueroa et al. 2016, 2010; Jaime et al. 2022; Sanchez‐Gonzalez et al. 2013), whereas combinatorial therapies included L‐citrulline + L‐arginine (Figueroa et al. 2014) or L‐citrulline + glutathione (Figueroa et al. 2023). Dosages ranged from 2 to 11 g/day, delivered twice daily in four studies (Figueroa et al. 2016, 2023, 2010; Jaime et al. 2022) and thrice daily in two (Figueroa et al. 2014; Sanchez‐Gonzalez et al. 2013). Intervention durations spanned 2 weeks (Figueroa et al. 2016; Jaime et al. 2022; Sanchez‐Gonzalez et al. 2013), 4 weeks (Figueroa et al. 2023, 2010), and 6 weeks (Figueroa et al. 2014). Five trials implemented cold pressor tests (CPT) by immersing extremities in 1°C–4°C water for 2–3 min (Figueroa et al. 2016, 2023, 2010, 2014; Jaime et al. 2022). One study exposed participants to a 4°C environmental chamber for 30 min before measuring resting BP, followed by 3‐min isometric handgrip exercises and post‐rest BP assessment, yielding dual datasets (Sanchez‐Gonzalez et al. 2013). Similarly, dual intervention arms (L‐citrulline alone and with glutathione) in one study generated two datasets (Figueroa et al. 2023).

### 3.3 Meta‐Analysis Results

#### 3.3.1 Effect of L‐Citrulline Supplementation on Brachial and Aortic Systolic Blood Pressure in Cold Environments

**FIGURE 2:**
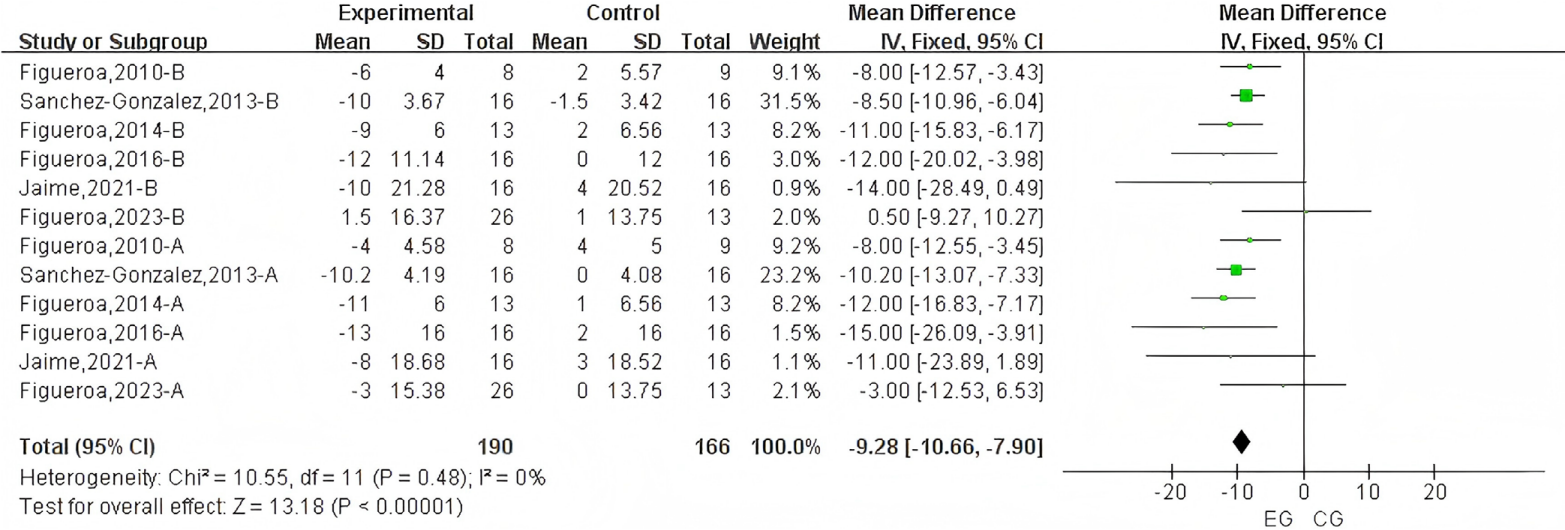
Forest plot of the effects of L‐Citrulline intake on brachial and aortic systolic blood pressure under cold exposure. A, brachial blood pressure; B, aortic blood pressure; EG, experimental group; CG, control group.

A total of 12 datasets were included in the meta‐analysis (Figure 2). Results demonstrated that L‐citrulline supplementation significantly reduced SBP under cold exposure (WMD = −9.28 mmHg, 95% CI: −10.66 to −7.90, *p* < 0.001), with moderate heterogeneity (*I* ^2^ = 0%). Sensitivity analysis, conducted by sequentially excluding individual studies, revealed no substantial impact on the overall results. Egger's test indicated no significant publication bias (*p* > 0.1).

#### 3.3.2 Effect of L‐Citrulline Supplementation on Brachial and Aortic Diastolic Blood Pressure in Cold Environments

**FIGURE 3:**
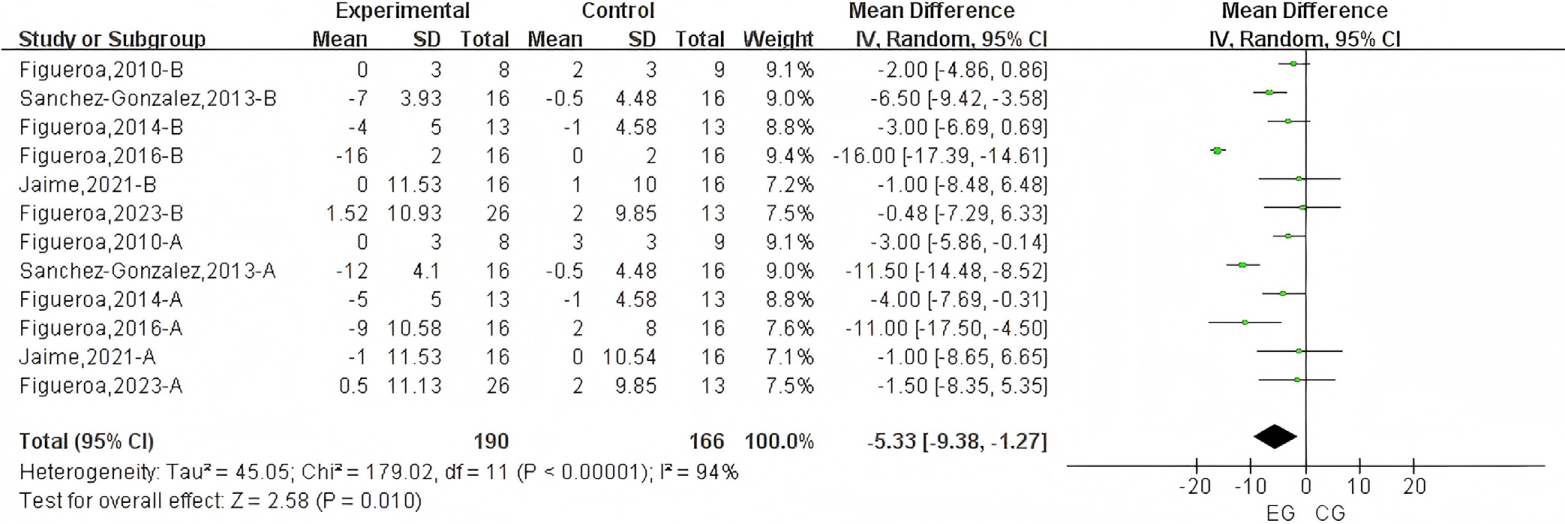
Forest plot of the effects of L‐Citrulline intake on brachial and aortic diastolic blood pressure under cold exposure. A, brachial blood pressure; B, aortic blood pressure; EG, experimental group; CG, control group.

A total of 12 datasets were included in the meta‐analysis (Figure 3). The results demonstrated that L‐citrulline intake in cold environments significantly reduced DBP (WMD = −5.33 mmHg, 95% CI: −9.38 to −1.27, *p* = 0.01), with substantial heterogeneity observed (*I* ^2^ = 94%). Sensitivity analysis through sequential removal of individual studies revealed that no single study substantially altered the overall results. Additionally, Egger's test indicated significant publication bias among the included studies (*p* = 0.007).

#### 3.3.3 Subgroup Analysis Results

**FIGURE 4:**
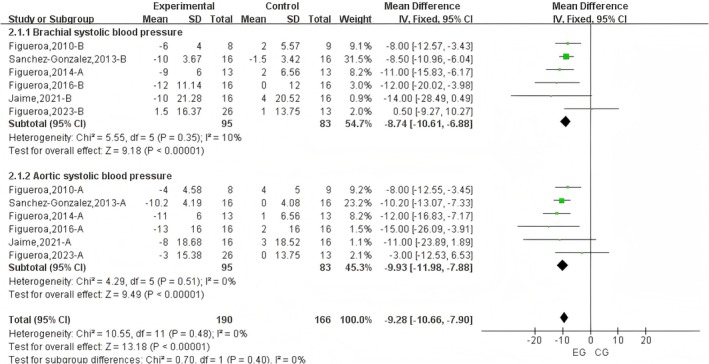
Subgroup analysis forest plot of the effects of L‐Citrulline intake on systolic blood pressure under cold exposure. A, brachial blood pressure; B, aortic blood pressure; EG, experimental group; CG, control group.

**FIGURE 5:**
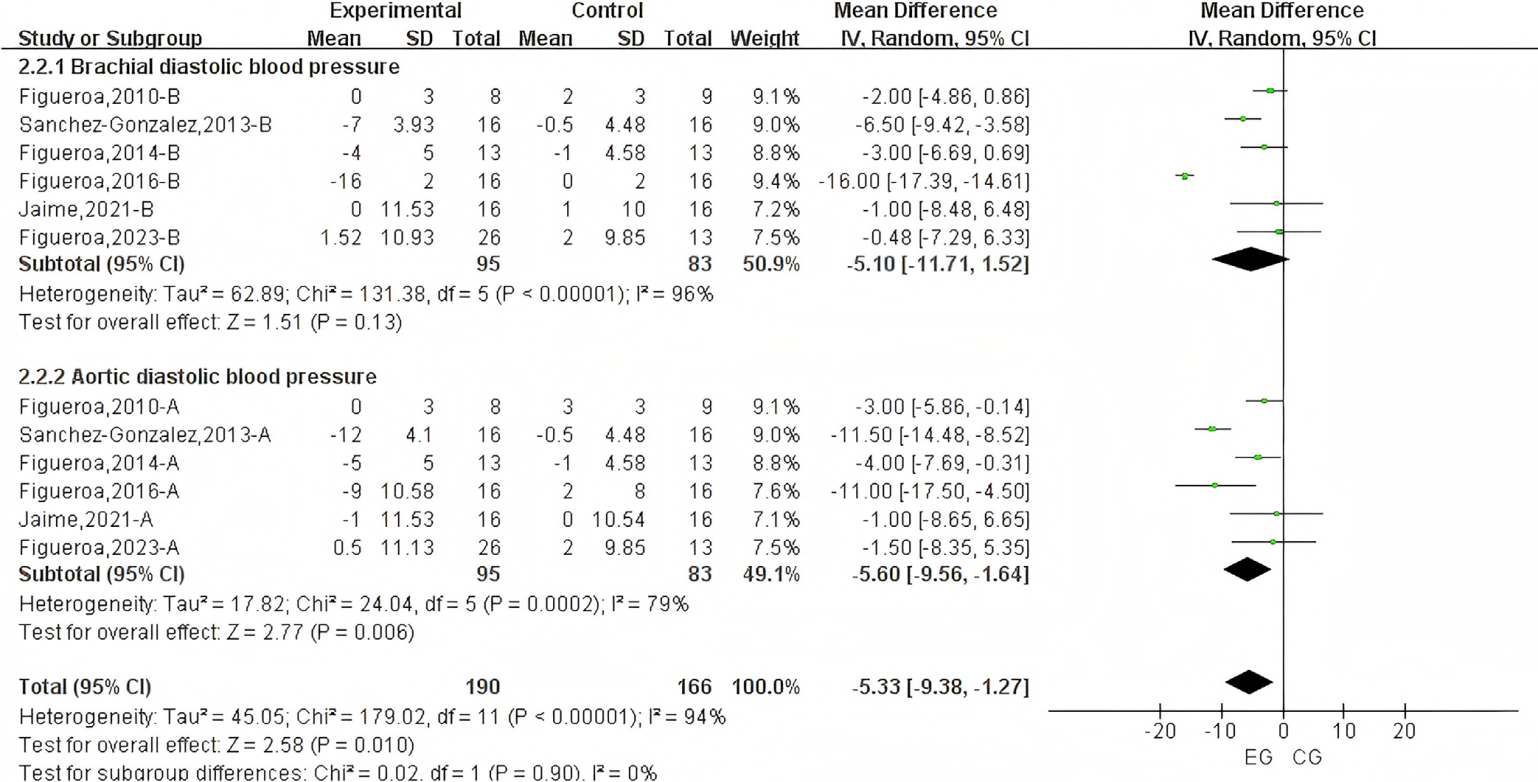
Subgroup analysis forest plot of the effects of L‐citrulline intake on diastolic blood pressure under cold exposure. A, brachial blood pressure; B, aortic blood pressure; EG, experimental group; CG, control group.

A subgroup analysis of SBP and DBP was conducted (Figures 4 and 5). The results demonstrated that L‐citrulline supplementation under cold conditions significantly reduced brachial artery SBP (WMD = −8.74 mmHg, 95% CI: −10.61 to −6.88, *p* < 0.001) and aortic SBP (WMD = −9.93 mmHg, 95% CI: −11.98 to −7.88, *p* < 0.001). Furthermore, L‐citrulline intake in cold environments significantly lowered aortic DBP (WMD = −5.60 mmHg, 95% CI: −9.56 to −1.64, *p* < 0.001). However, no significant reduction was observed in brachial artery DBP (WMD = −5.10 mmHg, 95% CI: −11.71 to 1.52, *p* = 0.13).

### 3.4 Risk of Bias and Literature Quality Assessment Results

The risk of bias assessment showed that for all included studies, the domains of Randomization Process, Missing Outcome Data, and Measurement of the Outcome were rated as low risk; one study had “some concerns” in Deviations from the Intended Interventions (Sanchez‐Gonzalez et al. 2013), and one study was rated “high risk” in Selection of the Reported Result (Figueroa et al. 2016). Overall, four studies were classified as “low risk” (Figueroa et al. 2023, 2010, 2014; Jaime et al. 2022), while two others were rated “some concerns”(Sanchez‐Gonzalez et al. 2013) and “high risk” (Figueroa et al. 2016) respectively. Literature quality assessment indicated that three studies scored 4 point (Figueroa et al. 2016, 2014; Sanchez‐Gonzalez et al. 2013), three studies scored 5 (Figueroa et al. 2010), 6 (Jaime et al. 2022), and 7 points (Figueroa et al. 2023), and all were deemed high‐quality. Detailed results are summarized in Appendix S1.

### 3.5 Publication Bias Test Results

**FIGURE 6:**
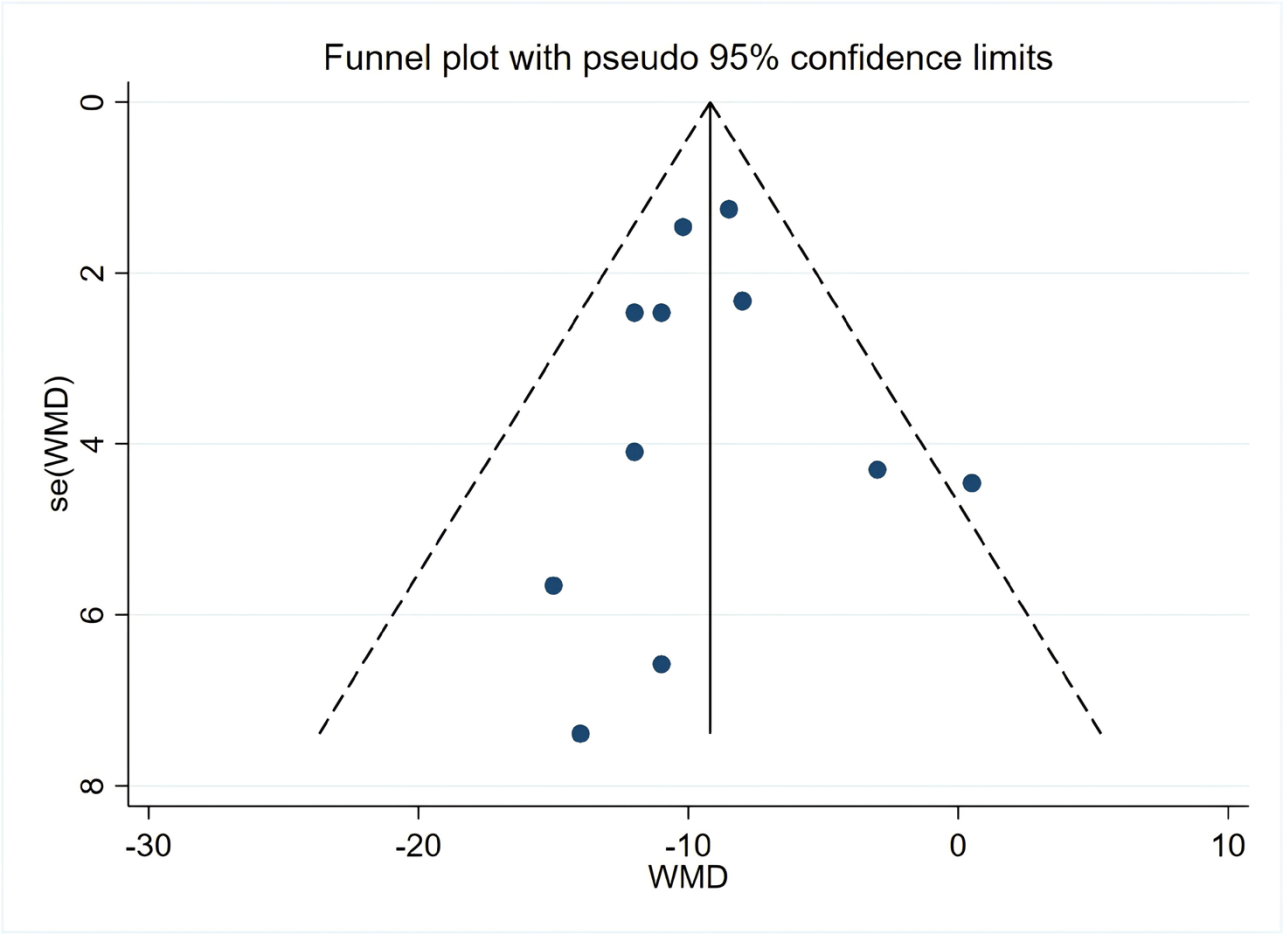
Funnel plot of publication bias for systolic blood pressure.

**FIGURE 7:**
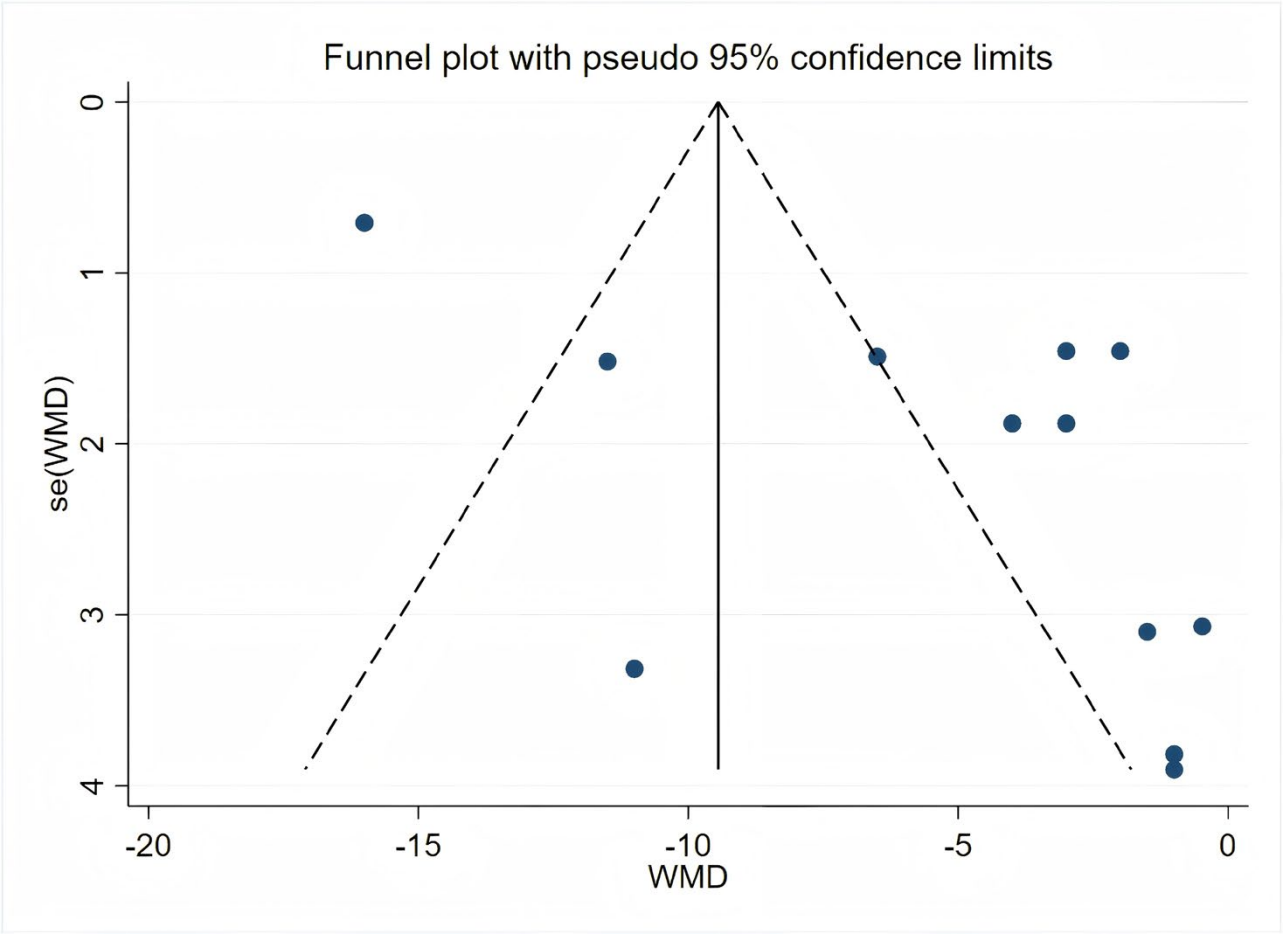
Funnel plot of publication bias for diastolic blood pressure.

The data of SBP and DBP were imported into Stata 17.0 to draw funnel plots (Figures 6 and 7) for visual assessment of publication bias. Concurrently, Egger's test was employed to quantitatively evaluate publication bias. The results indicated no significant publication bias for SBP results (*p* > 0.1). However, publication bias was detected for DBP results (*p* = 0.007). Further adjustment using the trim‐and‐fill method indicated that the pooled effect size for DBP remained statistically significant; however, the results suggest that potential bias might have attenuated the DBP effect size. The trim‐and‐fill results are provided in Appendix S1.

## 4 Discussion

This study represents the first systematic review and meta‐analysis investigating the effects of L‐citrulline supplementation on BP in cold environments. A total of 6 relevant RCTs involving 162 participants were included. The findings demonstrate that L‐citrulline supplementation in cold conditions significantly reduces both SBP and DBP. Subgroup analysis revealed significant reductions in brachial SBP, aortic SBP, and aortic DBP. Although brachial DBP reduction did not reach statistical significance, a significant decreasing trend was observed overall. These results suggest that L‐citrulline supplementation may serve as a potential nutritional intervention for blood pressure management in cold environments.

There is a significant negative correlation between BP and environmental temperature. Under cold conditions, BP markedly increases, with this effect being particularly pronounced in the elderly (Achebak et al. 2019; Bunker et al. 2016; Zheng et al. 2016), obese individuals, and patients with chronic diseases such as hypertension (Masajtis‐Zagajewska et al. 2021; Xu et al. 2024). This seasonal BP fluctuation significantly increases the risk of cardiovascular and cerebrovascular events (Khraishah et al. 2022; Stewart et al. 2017). This study confirms that L‐citrulline can effectively mitigate cold‐induced BP elevation through its unique pharmacological mechanisms, significantly reducing SBP (−9.28 mmHg, *p* < 0.001) and DBP (−5.33 mmHg, *p* = 0.01) in cold environments. L‐citrulline supplementation can increase L‐arginine levels (Schwedhelm et al. 2008; Waugh et al. 2001), which, as a precursor to NO, can promote NO production through the endothelial nitric oxide synthase (eNOS) pathway (Tousoulis et al. 2012; Vallance and Chan 2001). Not only does it directly dilate blood vessels by activating the cyclic guanosine monophosphate (cGMP) pathway, but it also offers multiple benefits such as inhibiting sympathetic activity, improving arterial compliance, and exerting anti‐inflammatory and antioxidant effects (Graham et al. 2018; Kleschyov et al. 2023; Leo et al. 2017). Regardless of the mechanisms, this study demonstrates that L‐citrulline can improve BP elevation caused by cold environments. This finding establishes its significant clinical value as a non‐pharmacological intervention strategy for BP management in cold conditions, providing a safe and feasible nutritional intervention plan for individuals at high cardiovascular risk during cold seasons. Additionally, all included clinical trials demonstrated good participant compliance with L‐citrulline supplementation and no reported drug‐related adverse events. Together with previous safety research evidence (Oketch‐Rabah et al. 2016; Romero et al. 2006; Smith et al. 2006), it is recommended that L‐citrulline be considered as an individualized dietary supplementation strategy for managing high BP in cold regions. Based on these findings, we advocate for increased intake of foods rich in L‐citrulline, such as watermelon, while enhancing seasonal BP monitoring and protective education for high‐risk groups, thereby effectively reducing health risks associated with cold‐induced BP elevation.

Subgroup analysis shows that L‐citrulline significantly reduces aortic SBP/DBP and brachial SBP (both *p* < 0.05), but the improvement in brachial DBP did not reach statistical significance (−5.10 mmHg, *p* = 0.13). Notably, after excluding the study by Figueroa et al. (2016), heterogeneity significantly decreased (*I* ^*2*^ from 94% to 50%), and the reduction in brachial DBP reached statistical significance (*p* = 0.01), suggesting that heterogeneity among the literature may have affected the initial results. Considering the strong correlation between aortic BP and cardiovascular prognosis, L‐citrulline's preferential improvement in central BP has greater clinical significance, while the consistent trend toward reduced brachial DBP (despite not achieving significance initially) remains noteworthy. The statistical results of this study exhibit certain heterogeneity, particularly in the DBP analysis of the aorta and brachial artery, where high *I* ^*2*^ values and publication bias were observed. This may be related to factors such as differences in subject characteristics across included studies, inconsistent oral dosages, and whether other nutritional supplements were co‐administered orally. Additionally, the trim‐and‐fill method was employed to estimate and correct for missing studies. The results showed that the pooled effect size for DBP remained statistically significant after correction, and no substantial impact of publication bias on the main conclusions was found, indicating that the DBP statistical results are relatively robust. Future research needs to expand sample sizes and adopt standardized measurement methods. Meanwhile, it is recommended to conduct cross‐sectional comparative studies of different dosages, establish unified dosing protocols, and use standardized blood pressure measurement equipment and procedures to provide conditions for subsequent meta‐analyses to explore different subgroup effects and sources of heterogeneity.

### 4.1 Limitations

This study has the following main limitations: First, most of the included studies employed CPT as an acute cold stimulus intervention, while lacking assessment of sustained BP responses in individuals under long‐term natural cold environments, which limits the practical application value of the research findings. Second, high heterogeneity may affect the robustness of the results. Third, the Egger test indicated publication bias regarding DBP (*p* = 0.007), which impacts the strength of evidence regarding the antihypertensive effect of L‐citrulline. Fourth, the limited number of included studies restricted the feasibility of subgroup analyses, making it challenging to explore the potential impacts of different population characteristics (e.g., age, baseline BP levels), intervention protocols (e.g., dosage, intervention duration), or environmental factors (e.g., intensity of cold exposure) on the antihypertensive effects of L‐citrulline. Finally, some of the studies included in this analysis employed combined intervention protocols of L‐citrulline with other ingredients (e.g., L‐arginine) (Figueroa et al. 2023; Sanchez‐Gonzalez et al. 2013). Therefore, the observed effects cannot completely exclude the potential contribution of these coexisting components. Although sensitivity analysis (provided in Appendix S1) showed that the robustness of the overall conclusions did not fundamentally change after excluding these combined intervention studies, this still, to some extent, limits the certainty of attributing the blood pressure‐lowering effects solely to L‐citrulline alone.

## 5 Conclusions

This study aimed to investigate whether L‐citrulline supplementation could counteract the adverse effects of cold environment on individual BP. The results demonstrated that L‐citrulline supplementation effectively alleviated the BP elevation response induced by cold exposure. Given that optimal management strategies for seasonal blood pressure variations require further research (Stergiou et al. 2020), and considering the unique antihypertensive effects of L‐citrulline in cold environments, future studies should design larger clinical trials to systematically observe dynamic changes in individual BP following L‐citrulline supplementation under long‐term cold exposure conditions to thoroughly elucidate its antihypertensive mechanisms and clinical application value.

## Author Contributions

P.L., J.C., K.L., and J.Z. conceived and designed this study. P.L., J.C., and K.L. wrote and analyzed the manuscript. J.Z. guided P.L., J.C., and K.L. in revising the article.

## Funding

This study was supported by the Fundamental Research Funds for the Central Universities (2024CDJSKXYTY03).

## Conflicts of Interest

The authors declare no conflicts of interest.

## Supporting information


**Table S1:** Risk of bias assessment using RoB 2.
**Table S2:** literature quality assessment.
**Table S3:** Search strategy.

## Data Availability

The data are real, reliable, and available. Supporting Information have been submitted. Supporting Information is available in the supplementary section.
