# Supplementary material for: Effect of L‐Citrulline Intake on Blood Pressure in Cold Environments: A Systematic Review and Meta‐Analysis of Randomized Controlled Trials
Source: Food Sci Nutr. 2026 Mar 6;14(3):e71603. doi: 10.1002/fsn3.71603 (PMC12965904; doi:10.1002/fsn3.71603)
Supplement: Supplementary file 1 — Table S1: Risk of bias assessment using RoB 2. Table S2: literature quality assessment. Table S3: Search strategy. [file FSN3-14-e71603-s001.docx]

**Supplementary Table 1. Risk of Bias Assessment using RoB 2**

| **Study lD** | **Randomisation process** | **Deviations from the intended interventions** | **Mising outcome data** | **Measurement of the outcome** | **Selection of the reported result** | **Overall risk-of-bias judgment** |
| --- | --- | --- | --- | --- | --- | --- |
| Figueroa,2010 | Low risk | Low risk | Low risk | Low risk | Low risk | Low risk |
| Sanchez-Gonzalez,2012 | Low risk | Some concerns | Low risk | Low risk | Low risk | Some concerns |
| Figueroa, 2014 | Low risk | Low risk | Low risk | Low risk | Low risk | Low risk |
| Figueroa, 2016 | Low risk | Low risk | Low risk | Low risk | High risk | High risk |
| Jaime，2022 | Low risk | Low risk | Low risk | Low risk | Low risk | Low risk |
| Figuero, 2023 | Low risk | Low risk | Low risk | Low risk | Low risk | Low risk |

**Supplementary Table 2. literature quality assessment**

| **Study lD** | **Random sequence production** | **Allocation concealment** | **Blinding method** | **Withdrawal** | **Total score of Jadad scale** |
| --- | --- | --- | --- | --- | --- |
| Figueroa,2010 | 2 | 1 | 2 | 0 | 5 |
| Sanchez-Gonzalez,2012 | 2 | 1 | 1 | 0 | 4 |
| Figueroa, 2014 | 2 | 1 | 1 | 0 | 4 |
| Figueroa, 2016 | 2 | 1 | 1 | 0 | 4 |
| Jaime，2022 | 2 | 1 | 2 | 1 | 6 |
| Figuero, 2023 | 2 | 2 | 2 | 1 | 7 |

**Supplementary Table 3. Search strategy**

| **1.PubMed: 9** |
| --- |
| Set1:("Citrulline"[Mesh]) OR ((citrulline malate[Title/Abstract]) OR (l-citrulline[Title/Abstract])) |
| Set2:("Cold Temperature"[Mesh]) OR ((((((((((chill temperature[Title/Abstract]) OR (low temperature[Title/Abstract])) OR (cold[Title/Abstract])) OR (chilly[Title/Abstract])) OR (frigid[Title/Abstract])) OR (cold conditions[Title/Abstract])) OR (frigid conditions[Title/Abstract])) OR (icy conditions[Title/Abstract])) OR (cold pressor test[Title/Abstract])) OR (CPT[Title/Abstract])) |
| Set3: ("Blood Pressure"[Mesh]) OR (((((((((((((((((((((((((((((BP[Title/Abstract]) OR (diastolic blood pressure[Title/Abstract])) OR (DBP[Title/Abstract])) OR (systolic blood pressure[Title/Abstract])) OR (SBP[Title/Abstract])) OR (high blood pressure[Title/Abstract])) OR (HBP[Title/Abstract])) OR (elevated blood pressure[Title/Abstract])) OR (hypertension[Title/Abstract])) OR (hypertensive[Title/Abstract])) OR (blood pressure reduction[Title/Abstract])) OR (rest blood pressure[Title/Abstract])) OR (mean blood pressure[Title/Abstract])) OR (blood pressure monitoring[Title/Abstract])) OR (BPM[Title/Abstract])) OR (ambulatory blood pressure[Title/Abstract])) OR (ABP[Title/Abstract])) OR (ambulatory blood pressure monitoring[Title/Abstract])) OR (ABPM[Title/Abstract])) OR (automated office blood pressure[Title/Abstract])) OR (AOBP[Title/Abstract])) OR (home blood pressure monitoring[Title/Abstract])) OR (HBPM[Title/Abstract])) OR (blood pressure management[Title/Abstract])) OR (resting blood pressure[Title/Abstract])) OR (daytime blood pressure[Title/Abstract])) OR (nighttime blood pressure[Title/Abstract])) OR (24 hours blood pressure[Title/Abstract])) OR (24-h blood pressure[Title/Abstract])) |
| Set4:(((((random[Title/Abstract]) OR (randomized[Title/Abstract])) OR (randomly[Title/Abstract])) OR (randomised[Title/Abstract])) OR (randomized controlled trial[Title/Abstract])) OR (RCT[Title/Abstract]) |
| Set5: #1 AND #2 AND #3 AND #4 |
|  |
| **2.Cochrane：18** |
| Set1:MeSH descriptor: [Citrulline] in all MeSH products |
| Set2:MeSH descriptor: [Cold Temperature] explode all trees |
| Set3:MeSH descriptor: [Blood Pressure] explode all trees |
| Set4:(citrulline malate):ti,ab,kw or (l-citrulline) |
| Set5:(chill temperature):ti,ab,kw or (low temperature):ti,ab,kw or (cold):ti,ab,kw or (chilly):ti,ab,kw or (frigid):ti,ab,kw or (cold conditions):ti,ab,kw or (frigid conditions):ti,ab,kw or (icy conditions):ti,ab,kw or (cold pressor test):ti,ab,kw or (CPT) |
| Set6:(BP):ti,ab,kw or (diastolic blood pressure):ti,ab,kw or (DBP):ti,ab,kw or (systolic blood pressure):ti,ab,kw or (SBP):ti,ab,kw or (high blood pressure):ti,ab,kw or (HBP):ti,ab,kw or (elevated blood pressure):ti,ab,kw or (hypertension):ti,ab,kw or (hypertensive):ti,ab,kw or (blood pressure reduction):ti,ab,kw or (rest blood pressure):ti,ab,kw or (mean blood pressure):ti,ab,kw or (blood pressure monitoring):ti,ab,kw or (BPM):ti,ab,kw or (ambulatory blood pressure):ti,ab,kw or (ABP):ti,ab,kw or (ambulatory blood pressure monitoring):ti,ab,kw or (ABPM):ti,ab,kw or (automated office blood pressure):ti,ab,kw or (AOBP):ti,ab,kw or (home blood pressure monitoring):ti,ab,kw or (HBPM):ti,ab,kw or (blood pressure management):ti,ab,kw or (resting blood pressure):ti,ab,kw or (daytime blood pressure):ti,ab,kw or (nighttime blood pressure):ti,ab,kw or (24 hours blood pressure):ti,ab,kw or (24h blood pressure) |
| Set7: #1 OR #4 |
| Set8: #2 OR #5 |
| Set9: #3 OR #6 |
| Set10: #7 AND #8 AND #9 |
|  |
| **3.Embase：16** |
| Set1:'citrulline'/exp |
| Set2:'cold'/exp |
| Set3:'blood pressure'/exp |
| Set4:'citrulline malate':ab,ti OR 'l-citrulline' |
| Set5:'chill temperature':ab,ti OR 'low temperature':ab,ti OR 'cold':ab,ti OR 'chilly':ab,ti OR 'frigid':ab,ti OR 'cold conditions':ab,ti OR 'frigid conditions':ab,ti OR 'icy conditions':ab,ti OR 'cold pressor test':ab,ti OR 'cpt' |
| Set6:'bp':ab,ti OR 'diastolic blood pressure':ab,ti OR 'dbp':ab,ti OR 'systolic blood pressure':ab,ti OR 'sbp':ab,ti OR 'high blood pressure':ab,ti OR 'hbp':ab,ti OR 'elevated blood pressure':ab,ti OR 'hypertension':ab,ti OR 'hypertensive':ab,ti OR 'blood pressure reduction':ab,ti OR 'rest blood pressure':ab,ti OR 'mean blood pressure':ab,ti OR 'blood pressure monitoring':ab,ti OR 'bpm':ab,ti OR 'ambulatory blood pressure':ab,ti OR 'abp':ab,ti OR 'ambulatory blood pressure monitoring':ab,ti OR 'abpm':ab,ti OR 'automated office blood pressure':ab,ti OR 'aobp':ab,ti OR 'home blood pressure monitoring':ab,ti OR 'hbpm':ab,ti OR 'blood pressure management':ab,ti OR 'resting blood pressure':ab,ti OR 'daytime blood pressure':ab,ti OR 'nighttime blood pressure':ab,ti OR '24 hours blood pressure':ab,ti OR '24-h blood pressure' |
| Set7:'random':ab,ti OR 'randomized':ab,ti OR 'randomly':ab,ti OR 'randomised':ab,ti OR 'randomized controlled trial':ab,ti OR 'rct' |
| Set8: #1 OR #4 |
| Set9: #2 OR #5 |
| Set10: #3 OR #6 |
| Set11: |
|  |
| **4.Web of science：12** |
| Set1:((TS=(citrulline)) OR TS=(citrulline malate)) OR TS=(l-citrulline) |
| Set2:((((((((((TS=(Cold Temperature)) OR TS=(chill temperature)) OR TS=(low temperature)) OR TS=(cold)) OR TS=(chilly)) OR TS=(frigid)) OR TS=(cold conditions)) OR TS=(frigid conditions)) OR TS=(icy conditions)) OR TS=(cold pressor test)) OR TS=(CPT) |
| Set3:(((((((((((((((((((((((((((((TS=(Blood pressure)) OR TS=(BP)) OR TS=(diastolic blood pressure)) OR TS=(DBP)) OR TS=(systolic blood pressure)) OR TS=(SBP)) OR TS=(high blood pressure)) OR TS=(HBP)) OR TS=(elevated blood pressure)) OR TS=(hypertension)) OR TS=(hypertensive)) OR TS=(blood pressure reduction)) OR TS=(rest blood pressure)) OR TS=(mean blood pressure)) OR TS=(blood pressure monitoring)) OR TS=(BPM)) OR TS=(ambulatory blood pressure)) OR TS=(ABP)) OR TS=(ambulatory blood pressure monitoring)) OR TS=(ABPM)) OR TS=(automated office blood pressure)) OR TS=(AOBP)) OR TS=(home blood pressure monitoring)) OR TS=(HBPM)) OR TS=(blood pressure management)) OR TS=(resting blood pressure)) OR TS=(daytime blood pressure)) OR TS=(nighttime blood pressure)) OR TS=(24 hours blood pressure)) OR TS=(24-h blood pressure) |
| Set4:(((((TS=(random)) OR TS=(randomized)) OR TS=(randomly)) OR TS=(randomised)) OR TS=(randomized controlled trial)) OR TS=(RCT) |
| Set5: #1 AND #2 AND #3 AND #4 |


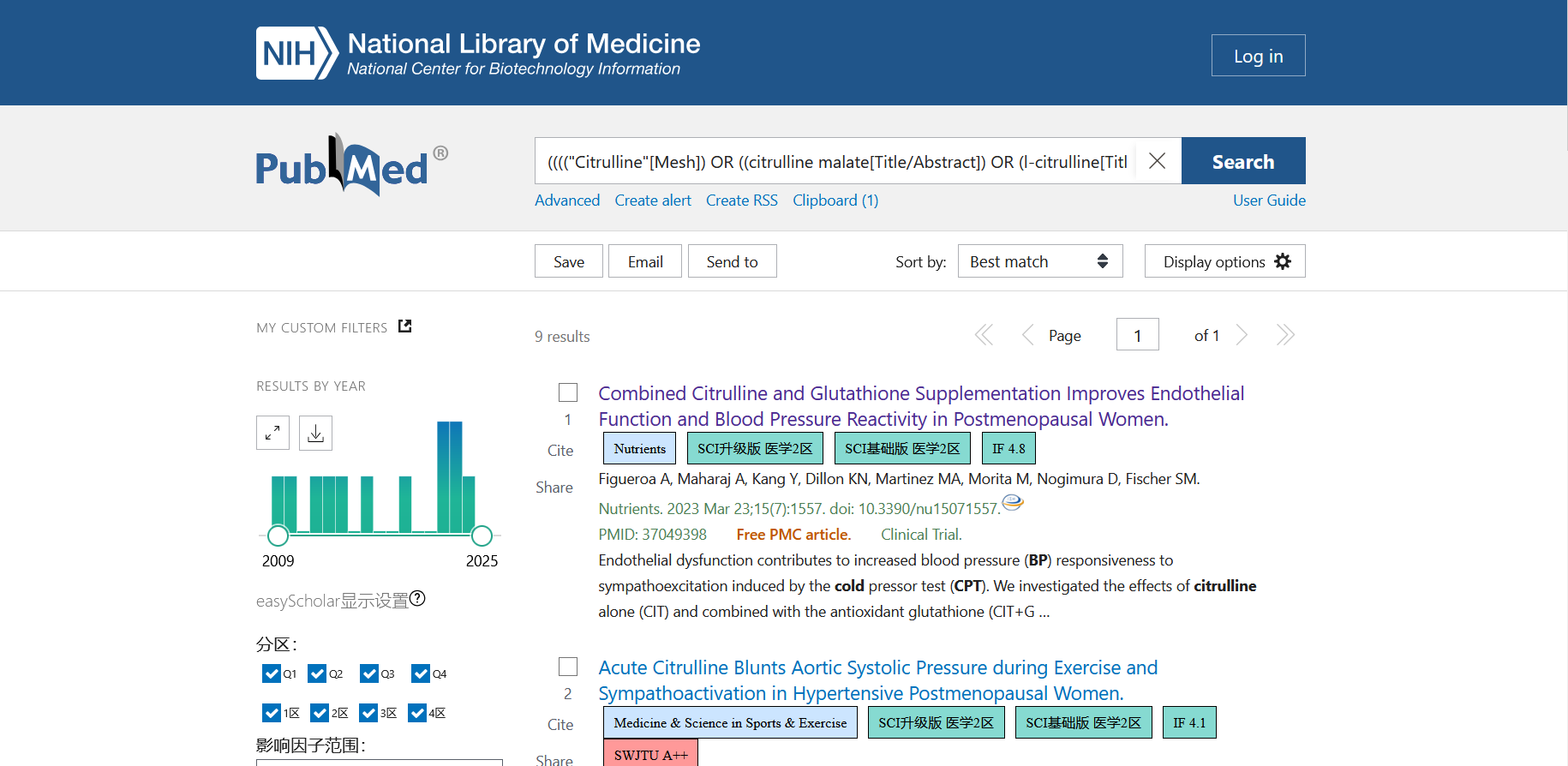


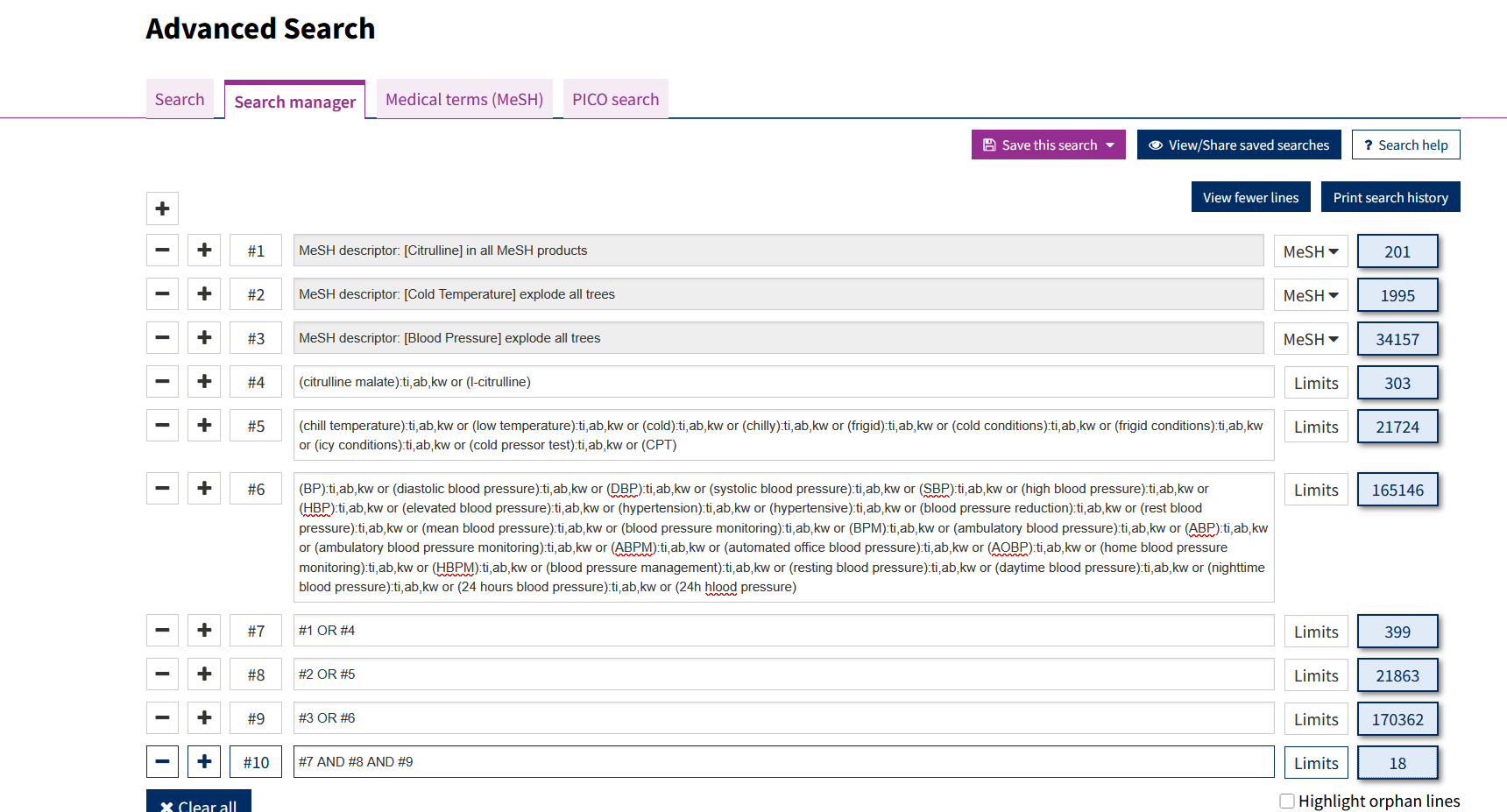


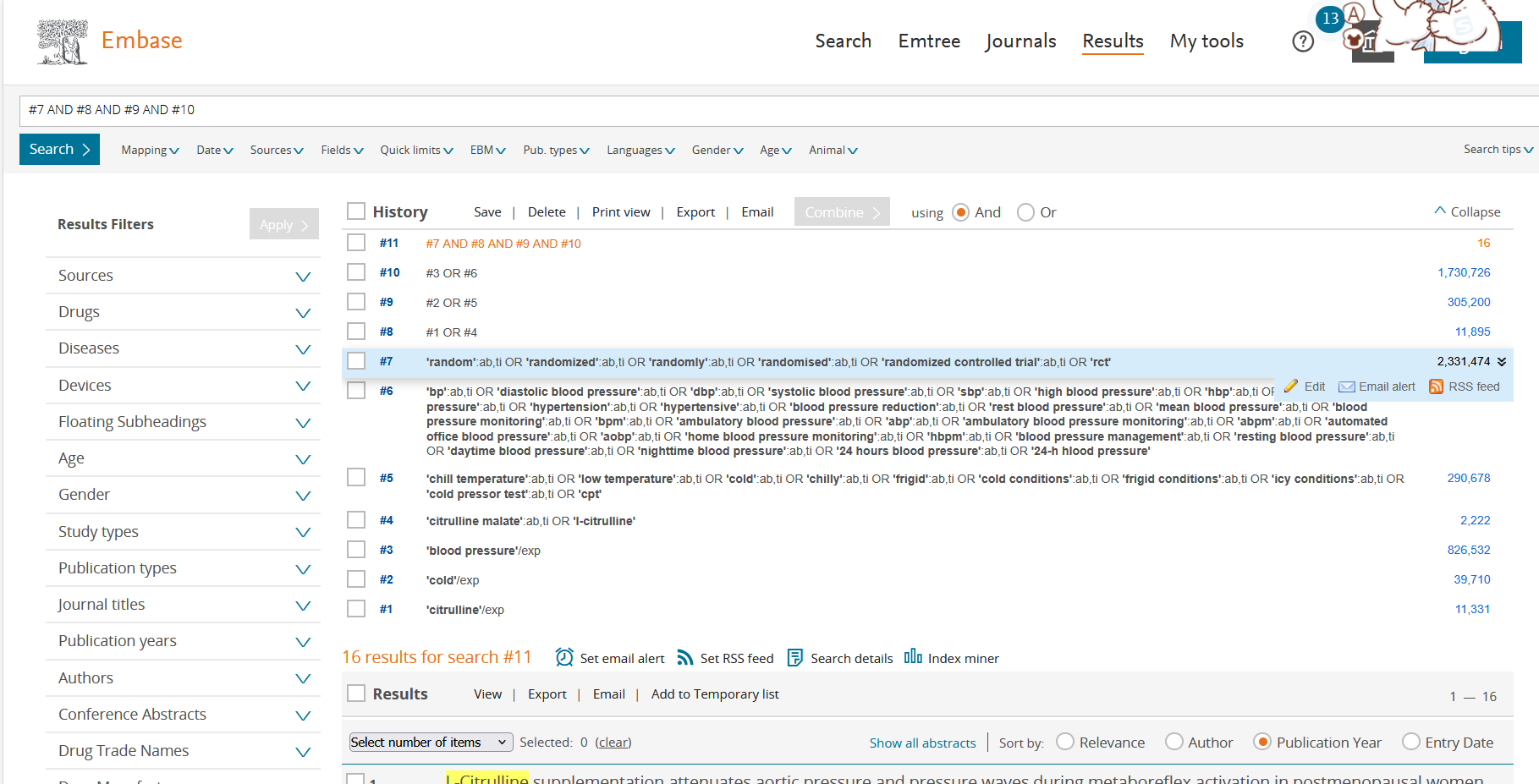


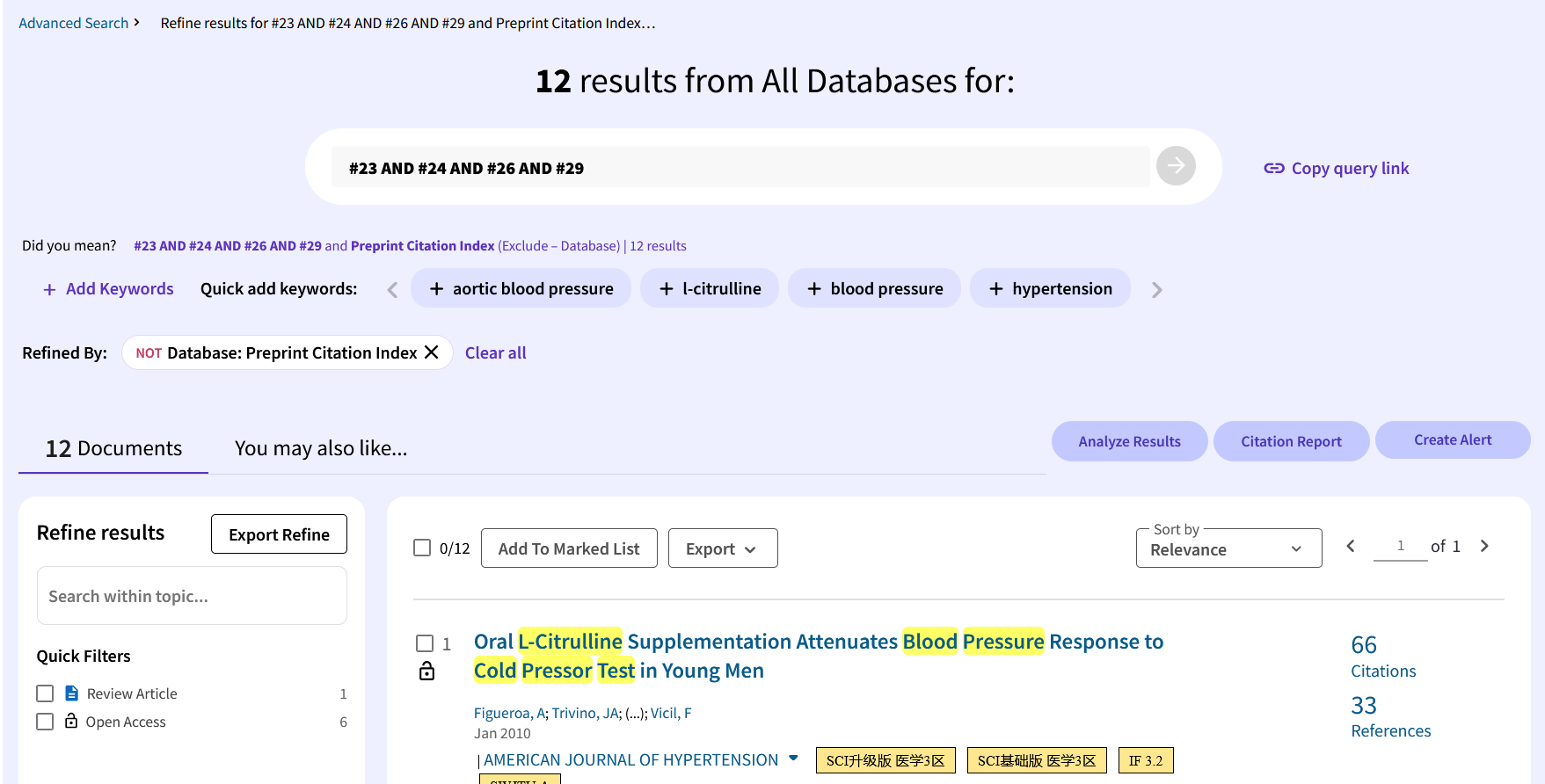


**
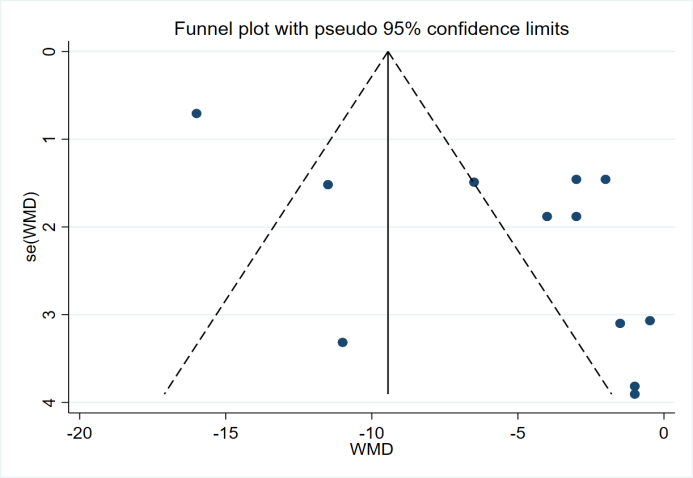
**

**Figure 1. Funnel plot for DBP**

**
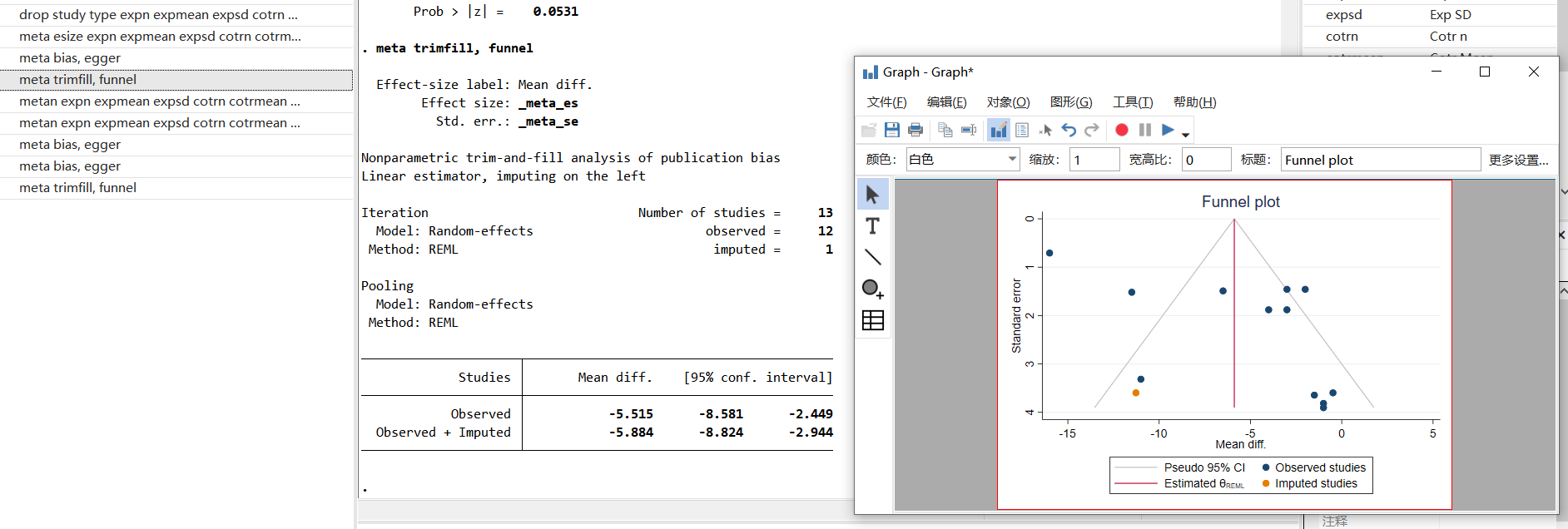
**

**Figure 2. Trim-and-fill plot**

**
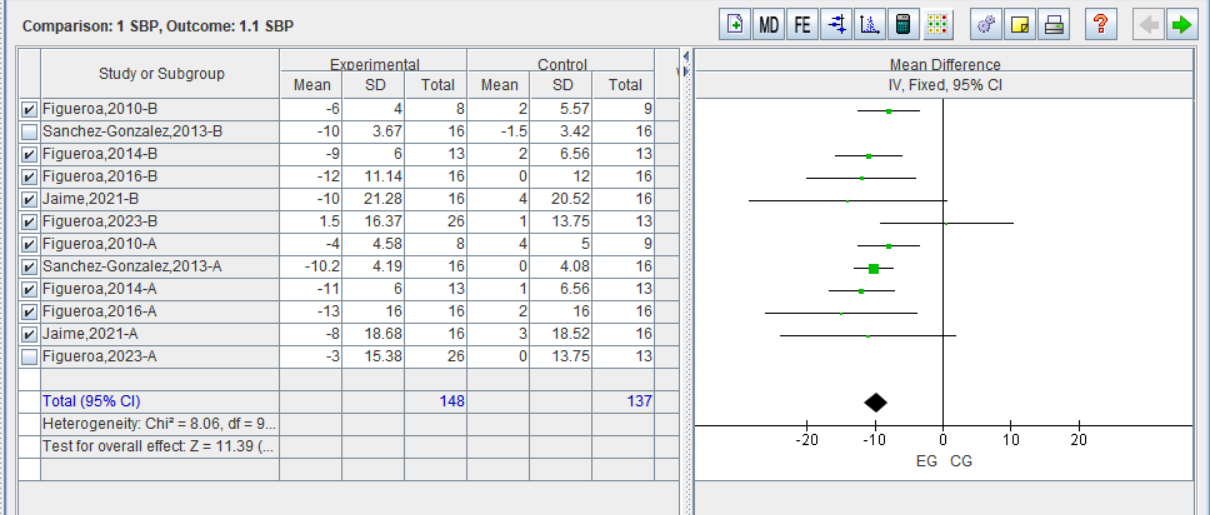
**

**Figure 3. Forest plot for subgroup analysis of SBP (after excluding the studies by Sanchez-Gonzalez et al. and Figueroa et al.)**

**
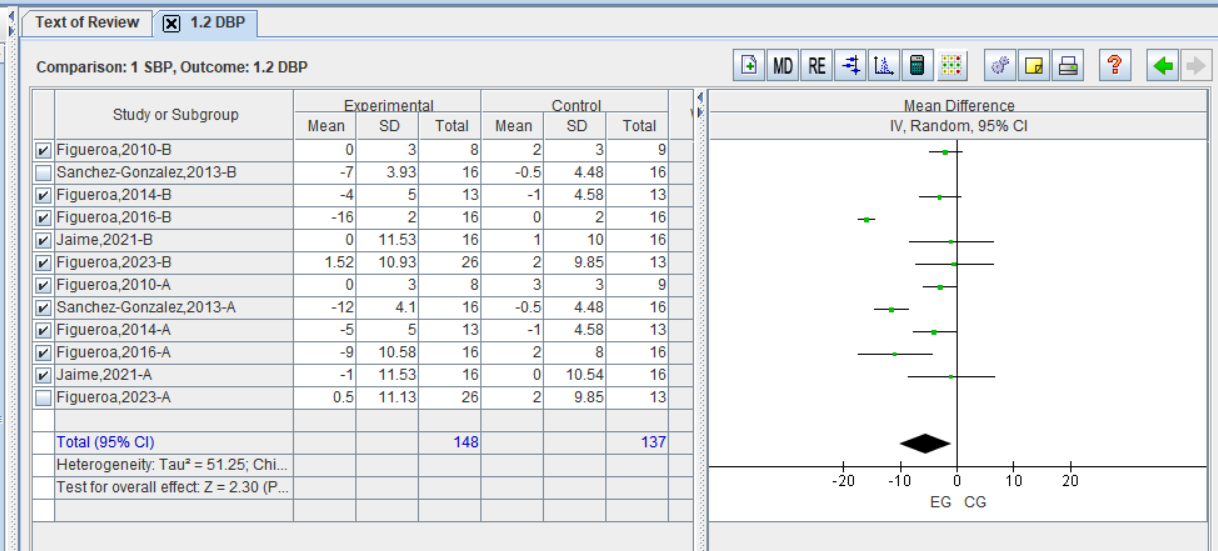
**

**Figure 4. Forest plot for subgroup analysis of DBP (after excluding the studies by Sanchez-Gonzalez et al. and Figueroa et al.)**
